# Exploring the potential mechanisms of Shiwei Hezi pill against nephritis based on the method of network pharmacology

**DOI:** 10.3389/fphar.2023.1178734

**Published:** 2023-06-09

**Authors:** Lei Wei

**Affiliations:** Department of Pharmacy, Northwest Women’s and Children’s Hospital, Xi’an, China

**Keywords:** network pharmacology, mechanisms, Shiwei Hezi pill, nephritis, molecular docking

## Abstract

**Objective:** We aimed to reveal the potential active ingredients, targets and pathways of Shiwei Hezi pill (SHP) in the treatment of nephritis based on systematic network pharmacology.

**Methods:** The online database was used to screen the common targets of SHP and nephritis, and the interaction between targets was analyzed. Gene Ontology (GO) functional annotation and Kyoto Encyclopedia of Genes and Genomes (KEGG) pathway enrichment analysis were performed using the Bioinformatics website. Molecular docking was carried out to verify the correlation between core ingredients and key targets. Cytoscape 3.6.1 was applied to perform protein-protein interactions (PPT) network construction and data visualization.

**Results:** A total of 82 active ingredients in SHP were screened, and 140 common targets of SHP and nephritis were obtained. Our results demonstrated that TNF, AKT1 and PTGS2 might be the key targets of SHP in the treatment of nephritis. GO enrichment analysis yielded 2163 GO entries (*p* < 0.05), including 2,014 entries of the biological process (BP) category, 61 entries of the cell composition (CC) category and 143 entries of the molecular function (MF) category. KEGG pathway enrichment analysis produced 186 signaling pathways (*p* < 0.05), involving the AGE-RAGE, IL-17and TNF signaling pathways. The results of molecular docking showed that three active ingredients in SHP (quercetin, kaempferol and luteolin) could effectively bind to the TNF, AKT1 and PTGS2 targets.

**Conclusion:** The effective active ingredients in SHP may regulate multiple signaling pathways through multiple targets, thereby exhibiting a therapeutic effect on nephritis.

## 1 Introduction

Nephritis is an immunity-mediated inflammatory response that has a great impact on human health (Fotioo, 1949; Kinoshita, Hirasawa et al., 1966). It belongs to a chronic kidney disease and has emerged as a major global health concern, accounting for approximately 69 percent of the total global disease burden. The mortality of nephritis is considerable and has increased 16.1 percent from 2006 to 2016. In China, around 119.5 million patients suffered chronic kidney disease, making it the second highest chronic disease after hypertension (254 million), and the number of patients with chronic kidney disease is higher than that with diabetes (113.9 million). Due to the unclear etiology and untypical early clinical symptoms of renal disease secondary to nephritis, coupled with the fact that some patients are skeptical of existing clinical treatments, research on drugs for the treatment of nephritis is urgently needed.

With the continuous development of Chinese medical practice, traditional Chinese medicine (TCM) is becoming increasingly recognized for its potential in treating chronic and complex diseases both at home and abroad (Cheung, 2011). In 2010, TCM products exported to Europe and the United States amounted US$ 2 billion and US$ 7.6 billion, respectively, and these figures are still growing (Cheung, 2011). Of course, TCM is also believed to play a vital role in the treatment of kidney-related diseases. For example, Ying Ding et al. have revealed the better curative effect of *Tripterygium wilfordii* combined with *Salvia miltiorrhiza* on children with allergic purpura nephritis (Ding, Zhang et al., 2019). Wan Yudang et al. have summarized the clinical characteristics of patients with heat shock nephritis, providing the most effective theoretical basis for TCM in terms of heat shock nephritis treatment (Dang, Yan et al., 2018). Overall, TCM has been studied to be effective in the treatment of kidney-related diseases, especially nephritis.

Shiwei Hezi pill (SHP) is a classic Tibetan medicine prescription commonly used to treat nephritis, which consists of ten species of medicinal herbs, including *Chebulae Fructus* (Hezi), *Carthami Flos* (Honghua), *Radixet Rhizoma Rubiae* (Zangqiancao), *Mountain alum leaf* (Shanfanye), *Swertia petiolata* (Zhangyacai), *Lithospermum erythrorhizon* (Zicaorong), *Canavaliae Semen* (Daodou), Alpinia Katsumadai Hayat (Doukou), *Slag breaking cream* (Zhaxungao), and *Sabina chinensis* (Yuanbai) (Table 1). The effectiveness of SHP in treating nephritis has been demonstrated in many studies. For instance, in a study by Ramala, nephritis patients treated with Yishen fossil granules were recruited for the control group, and patients treated with SHP were for the study group. By comparison, Tibetan medicine SHP exhibited high therapeutic efficiency and had a positive effect on alleviating the symptoms of nephritis (Ala, 2017). However, due to the complex formula of SHP, its specific mechanism in the treatment of nephritis remains unclear.

**TABLE 1:** A list of name categories for SHP.

Number	Herb name	
	Chinese spelling	Latin name
1	Hezi	Chebulae Fructus
2	Honghua	Carthami Flos
3	Zangqiancao	Radixet Rhizoma Rubiae
4	Shanfanye	Mountain alum leaf
5	Zhangyacai	Swertia petiolata
6	Zicaorong	Lithospermum erythrorhizon
7	Daodou	Canavaliae Semen
8	Doukou	Alpinia Katsumadai Hayat
9	Zhaxungao	Slag breaking cream
10	Yuanbai	Sabina chinensis

Nowadays, network pharmacology has been extensively adopted to elucidate the mechanism of TCM compounds and recipes in the treatment of diseases, and in other words, the mystery of TCM prescriptions in the treatment of complex diseases has been gradually unraveled. For example, Yue SJ et al. have explained the mechanism of Danggui-Honghua in the treatment of blood stasissyndrome by the systems pharmacology approach (Yue, Xin et al., 2017); Liu J et al. have revealed the therapeutic properties of Saffron formula in treating cardiovascular diseases based on systematic pharmacology dissection (Liu, Mu et al., 2016); Pang XC et al. have employed the virtual screening and network pharmacological methods to analyze the potential efficacy of Naodesheng formula in the treatment of Alzheimer’s disease (Pang, Kang et al., 2018); and similar scientific approaches were also performed by Xie W et al. to predict the anti-depressive effect of *Panax Notoginseng Saponins* (Xie, Meng et al., 2018). However, there are no in-depth studies which explore the specific mechanism of SHP in nephritis treatment by applying network pharmacological method.

**FIGURE 1:**
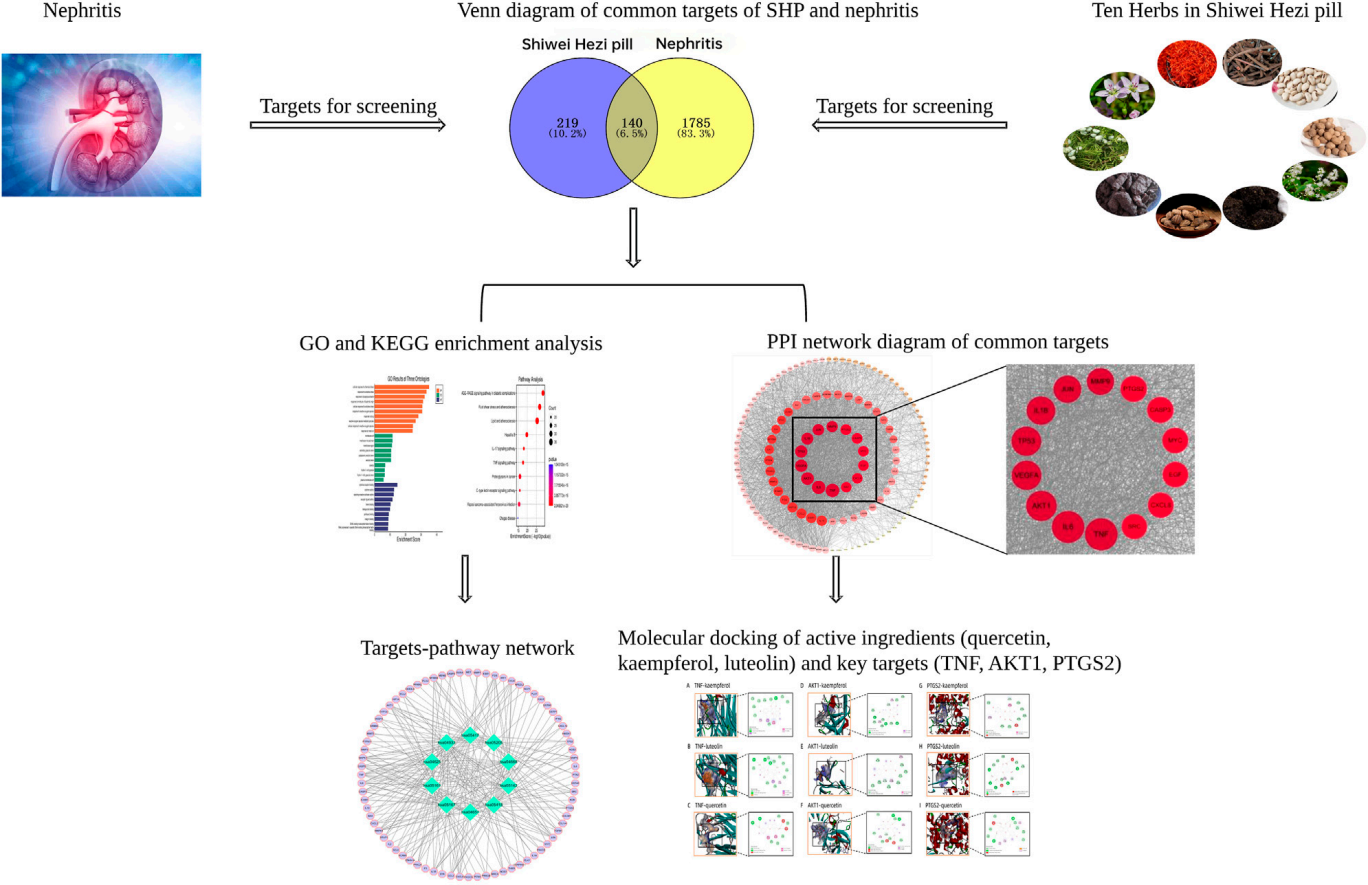
Network pharmacology flow chart of SHP in treating nephritis, including database preparation, PPI network construction, GO and KEGG pathway analyses, and molecular docking validation.

This study adopted network pharmacology method to identify the potential active ingredients, key targets and pathways of SHP in the treatment of nephritis, and molecular docking was carried out to investigate the interactions between selected key targets and active compounds. As shown in Figure 1, a schematic diagram of network pharmacological strategy was generated to determine the pharmacological mechanism of SHP in treating nephritis.

## 2 Methods

### 2.1 Acquisition of SHP ingredients

The chemical composition of each herb in SHP was obtained from free public databases, including TCMSP (http://ibts.hkbu.edu.hk/LSP/tcmsp.php) and TCMID (http://tcm.cmu.edu.tw) databases (Chen, 2011; Ru, Li et al., 2014). SHP ingredients with oral bioavailability (OB) ≥30% and drug-likeness (DL) ≥0.18 were then screened according to a number of relevant literature criteria. Ultimately, a data set of the potential active ingredients in SHP was constructed (Chandran, Mehendale et al., 2015; Kibble, Saarinen et al., 2015; Huang, Cheung et al., 2017).

### 2.2 Acquisition of SHP-related targets

The targets of the potential active ingredients in SHP were downloaded from the TCMSP database, and their duplicates were deleted. The UniProt (https://www.uniprot.org/) database was retrieved to annotate the target protein sequence, after that, the corresponding targets of the bioactive ingredients in SHP were obtained. We then established the ingredient-target data set by converting the target sites into gene names with the species limit to “*Homo sapiens*” by the DAVID database (https://david.ncifcrf.gov) (Dennis, Sherman et al., 2003).

### 2.3 Acquisition of nephritis-related targets

The nephritis-related targets were filtered by searching the PubMed (https://pubmed.ncbi.nlm.nih.gov/) (Amberger, Bocchini et al., 2015), OMIM (http://omim.org/) (Sayers, Agarwala et al., 2019), and GeneCard (https://www.genecards.org/) (Stelzer, Rosen et al., 2016) databases using “Nephritis” as a keyword. Afterwards, deduplication was performed, and a data set of nephritis-related targets was successfully created.

### 2.4 Acquisition of common targets of SHP and nephritis

The SHP and nephritis-related targets were imported into the Venny 2.1.0 online platform (https://bioinfogp.cnb.csic.es/tools/venny/) to construct a Venn diagram of common targets of SHP and nephritis.

### 2.5 Construction of protein-protein interaction (PPI) network

The data set of common targets of SHP and nephritis was imported into the STRING database Version 10.5 (https://cn.string-db.org/) with the species limit to “*H. sapiens*”. The PPI was obtained and saved as a “tsv” format file. Afterwards, the file was input into Cytoscape 3.6.1 to build a PPI network.

### 2.6 Gene Ontology (GO) and Kyoto Encyclopedia of Genes and Genomes (KEGG) pathway enrichment analyses

GO functional annotation and KEGG pathway analysis were performed via the Bioinformatics website (http://bioinformatics.com.cn/). By entering a list of target gene names and restricting the species to human, all target gene names were corrected to their official gene symbols. The top 15 and top 20 entries, including biological processes (BP), cellular components (CC) and molecular functions (MF), were selected for analysis with *p*-value as a screening criterion. The histograms and bubble charts were plotted to further explore the biological significance of common targets of SHP and nephritis.

### 2.7 Construction of network

In order to further investigate the mechanism action of SHP in nephritis treatment, the herb-ingredient, herb-ingredient-target-disease and target-pathway networks were established and visualized using Cytoscape 3.7.1, an open source software for visualizing complex networks (Su, Morris et al., 2014). In these networks, “nodes” represented herbs, compounds, targets, diseases or pathways, and “edges” represented the interactions between them.

### 2.8 Molecular docking

The most important active ingredients and action targets are selected for molecular docking. The files of 3D structures of compounds were downloaded in SDF format from the PubChem database, while the files of the 3D structures of targets were obtained in PDB format from the Protein Data Bank (PDB) database (https://www.rcsb.org/) (Chen, Chen et al., 2019). Using AutoDock, water molecules, hydrogen, and charges were removed, and the PDBQT format was saved. Subsequently, AutoDock Vina was used to performed molecular docking. Optimal docking score combinations were visualized by PyMOL 2.4.

## 3 Results

### 3.1 Bioactive ingredients in SHP

**FIGURE 2:**
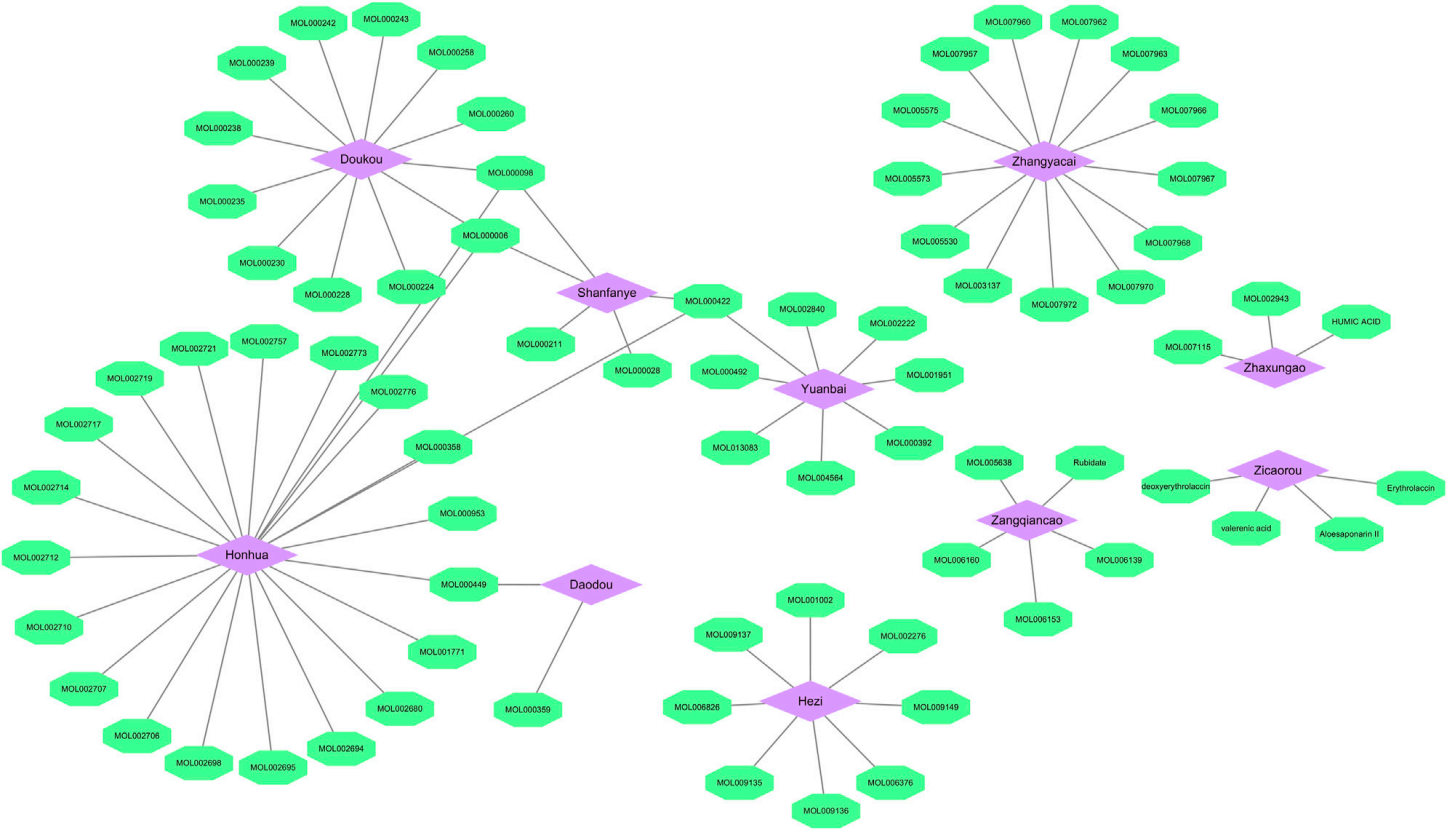
Herb-ingredient network. Purple represents the herb and green represents the active ingredient (Mol ID) of traditional Chinese medicine.

Combining TCMSP and TCMID databases with literature search, 82 bioactive ingredients (Table 2) of 10 herbs in SHP were obtained. Among them, the active ingredients of 5 herbs (*Chebulae Fructus*, *Carthami Flos*, *Canavaliae Semen*, *Alpinia Katsumadai Hayat*, and *S. petiolata*) were obtained from the TCMSP and TCMID databases, while the active ingredients of the remaining 5 herbs were identified through the literature search. Notably, despite madder diester and n-butanol with OB < 30% and DL < 0.18, they still showed a high content and potential anti-nephritis value, making them as active ingredients. Cytoscape software was then used to construct a compositional network diagram, and 75 active ingredients were gained after removing duplicate ingredients of this herb (Figure 2).

**TABLE 2:** The basic information list of chemical constituents in SHP.

Number	Herbs	Mol ID	Molecule name	MW	OB (%)	DL
1	Daodou	MOL000359	sitosterol	414.79	36.91	0.75
2	Daodou	MOL000449	Stigmasterol	412.77	43.83	0.76
3	Honghua	MOL001771	poriferast-5-en-3beta-ol	414.79	36.91	0.75
4	Honghua	MOL002680	Flavoxanthin	584.96	60.41	0.56
5	Honghua	MOL002694	4-[(E)-4-(3,5-dimethoxy-4-oxo-1-cyclohexa-2,5-dienylidene)but-2-enylidene]-2,6-dimethoxycyclohexa-2,5-dien-1-one	356.4	48.47	0.36
6	Honghua	MOL002695	lignan	458.55	43.32	0.65
7	Honghua	MOL002698	lupeol-palmitate	665.26	33.98	0.32
8	Honghua	MOL002706	Phytoene	545.04	39.56	0.5
9	Honghua	MOL002707	phytofluene	543.02	43.18	0.5
10	Honghua	MOL002710	Pyrethrin II	372.5	48.36	0.35
11	Honghua	MOL002712	6-Hydroxykaempferol	302.25	62.13	0.27
12	Honghua	MOL002714	baicalein	270.25	33.52	0.21
13	Honghua	MOL002717	qt_carthamone	286.25	51.03	0.2
14	Honghua	MOL002719	6-Hydroxynaringenin	288.27	33.23	0.24
15	Honghua	MOL002721	quercetagetin	318.25	45.01	0.31
16	Honghua	MOL002757	7,8-dimethyl-1H-pyrimido [5,6-g]quinoxaline-2,4-dione	242.26	45.75	0.19
17	Honghua	MOL002773	beta-carotene	536.96	37.18	0.58
18	Honghua	MOL002776	Baicalin	446.39	40.12	0.75
19	Honghua	MOL000358	beta-sitosterol	414.79	36.91	0.75
20	Honghua	MOL000422	kaempferol	286.25	41.88	0.24
21	Honghua	MOL000006	luteolin	286.25	36.16	0.25
22	Honghua	MOL000953	CLR	386.73	37.87	0.68
23	Honghua	MOL000098	quercetin	302.25	46.43	0.28
24	Honghua	MOL000449	Stigmasterol	412.77	43.83	0.76
25	Hezi	MOL001002	ellagic acid	302.2	43.06	0.43
26	Hezi	MOL002276	Sennoside E_qt	524.5	50.69	0.61
27	Hezi	MOL006376	7-Dehydrosigmasterol	414.79	37.42	0.75
28	Hezi	MOL006826	chebulic acid	356.26	72	0.32
29	Hezi	MOL009135	ellipticine	246.33	30.82	0.28
30	Hezi	MOL009136	Peraksine	310.43	82.58	0.78
31	Hezi	MOL009137	(R)-(6-methoxy-4-quinolyl)-[(2R,4R,5S)-5-vinylquinuclidin-2-yl]methanol	324.46	55.88	0.4
32	Hezi	MOL009149	Cheilanthifoline	325.39	46.51	0.72
33	Doukou	MOL000224	(4E,6E)-1,7-bis(3,4-dihydroxyphenyl)hepta-4,6-dien-3-one	326.37	33.06	0.31
34	Doukou	MOL000228	(2R)-7-hydroxy-5-methoxy-2-phenylchroman-4-one	270.3	55.23	0.2
35	Doukou	MOL000230	Pinocembrin	270.3	57.56	0.2
36	Doukou	MOL000235	1,7-diphenyl-3,5-dihydroxy-1-heptene	282.41	49.01	0.18
37	Doukou	MOL000238	1,7-diphenyl-5-hydroxy-6-hepten-3-one	280.39	32.65	0.18
38	Doukou	MOL000239	Jaranol	314.31	50.83	0.29
39	Doukou	MOL000242	7-O-Methyleriodictyol	302.3	56.56	0.27
40	Doukou	MOL000243	alpinolide peroxide	282.37	87.67	0.19
41	Doukou	MOL000258	dehydrodiisoeugenol	312.39	56.84	0.29
42	Doukou	MOL000260	5-[(2R,3R)-7-methoxy-3-methyl-5-[(E)-prop-1-enyl]-2,3-dihydrobenzofuran-2-yl]-1,3-benzodioxole	324.4	65.55	0.4
43	Doukou	MOL000006	luteolin	286.25	36.16	0.25
44	Doukou	MOL000098	quercetin	302.25	46.43	0.28
45	Zhangyacai	MOL003137	Leucanthoside	462.44	32.12	0.78
46	Zhangyacai	MOL005530	Hydroxygenkwanin	300.28	36.47	0.27
47	Zhangyacai	MOL005573	Genkwanin	284.28	37.13	0.24
48	Zhangyacai	MOL005575	Gentiacaulein	288.27	72.82	0.27
49	Zhangyacai	MOL007957	Swertiaperennin	288.27	96.85	0.27
50	Zhangyacai	MOL007960	8-hydroxy-1,2,6-trimethoxy-xanthone	302.3	77.13	0.3
51	Zhangyacai	MOL007962	1,7- dihydroxy-3,5-dimethoxy xanthone	288.27	103.37	0.27
52	Zhangyacai	MOL007963	1-hydroxy-2,3,5-trimethoxy-xanthone	302.3	101.06	0.3
53	Zhangyacai	MOL007966	1-Hydroxy-2,3,4,7-tetramethoxyxanthone	332.33	88.86	0.37
54	Zhangyacai	MOL007967	1-hydroxy-2,3,5,7-tetramethoxyxanthone	332.33	97.52	0.37
55	Zhangyacai	MOL007968	norbellidifolin	262.23	58.82	0.22
56	Zhangyacai	MOL007970	5,8-Dimethylbellidifolin	302.3	99.75	0.3
57	Zhangyacai	MOL007972	8-hydroxypinoresinal	374.42	71.09	0.55
58	Zangqiancao	MOL006160	Alizarin	240.22	32.67	0.19
59	Zangqiancao	MOL005638	Mollugin	284.33	42.34	0.26
60	Zangqiancao	—	Rubidate	—	—	—
61	Zangqiancao	MOL006139	1,3-dimethoxy-2-carboxyanthraquinone	312.29	102.89	0.33
62	Zangqiancao	MOL006153	2′-hydroxymollugin	302.35	40.5	0.29
63	Shanfanye	MOL000006	luteolin	286.25	36.16	0.25
64	Shanfanye	MOL000028	α-Amyrin	426.8	39.51	0.76
65	Shanfanye	MOL000211	Mairin	456.78	55.38	0.78
66	Shanfanye	MOL000422	kaempferol	286.25	41.88	0.24
67	Shanfanye	MOL000098	quercetin	302.25	46.43	0.28
68	Zhaxungao	MOL007115	manool	304.57	45.04	0.2
69	Zhaxungao	—	HUMIC ACID	—	—	—
70	Zhaxungao	MOL002943	1-Butanol	74.14	22.02	0
71	Zicaorong	—	valerenic acid	—	—	—
72	Zicaorong	—	Erythrolaccin	—	—	—
73	Zicaorong	—	deoxyerythrolaccin	—	—	—
74	Zicaorong	—	Aloesaponarin II	—	—	—
75	Yuanbaigao	MOL000422	kaempferol	286.25	41.88	0.24
76	Yuanbaigao	MOL013083	Skimmin (8CI)	324.31	38.35	0.32
77	Yuanbaigao	MOL000492	(+)-catechin	290.29	54.83	0.24
78	Yuanbaigao	MOL002840	Cryptopimaric acid	302.5	39.58	0.28
79	Yuanbaigao	MOL002222	sugiol	300.48	36.11	0.28
80	Yuanbaigao	MOL001951	Bergaptin	338.43	41.73	0.42
81	Yuanbaigao	MOL000392	formononetin	268.28	69.67	0.21
82	Yuanbaigao	MOL004564	Kaempferid	300.28	73.41	0.27

MW: molecule weight; OB(%): oral bioavailability; DL: drug-like properties.

### 3.2 Acquisition of common targets of SHP and nephritis

**FIGURE 3:**
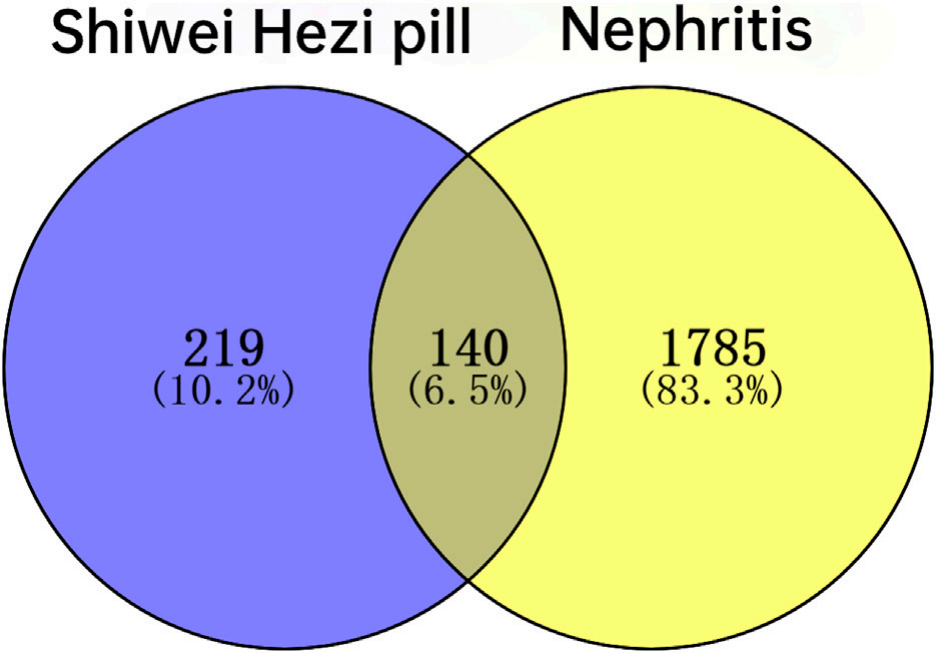
Venn diagram of the common targets of SHP and nephritis.

A total of 861 SHP-related targets were obtained based on the TCMSP database. And after the deduplication of the targets corresponding to 75 ingredients of SHP, we acquired 359 SHP-related targets. According to the Pubmed, OMIM and GeneCards databases, a total of 1925 targets related to nephritis were obtained. Ultimately, inputting 359 SHP targets and 1925 nephritis targets into Venny 2.1 software, a total of 140 targets common to both SHP and nephritis were found, and a Venn diagram was established (Table 3; Figure 3).

**TABLE 3:** A list of basic information about the common targets of SHP and nephritis.

Number	Target name	Gene symbol
1	Retinoic acid receptor RXR-alpha	*RXRA*
2	Prostaglandin G/H synthase 1	*PTGS1*
3	Prostaglandin G/H synthase 2	*PTGS2*
4	Urokinase-type plasminogen activator	*PLAU*
5	Estrogen receptor	*ESR1*
6	Nitric oxide synthase, inducible	*NOS2*
7	RAC-alpha serine/threonine-protein kinase	*AKT1*
8	Vascular endothelial growth factor A	*VEGFA*
9	72 kDa type IV collagenase	*MMP2*
10	Caveolin-1	*CAV1*
11	Transforming growth factor beta-1	*TGFB1*
12	E-selectin	*SELE*
13	Interleukin-6	*IL6*
14	Nitric oxide synthase, endothelial	*NOS3*
15	Plasminogen activator inhibitor 1	*SERPINE1*
16	Collagen alpha-1(I) chain	*COL1A1*
17	Cyclin-dependent kinase inhibitor 1	*CDKN1A*
18	Matrix metalloproteinase-9	*MMP9*
19	Interleukin-10	*IL10*
20	Tumor necrosis factor	*TNF*
21	Caspase-3	*CASP3*
22	Peroxisome proliferator-activated receptor gamma	*PPARG*
23	Intercellular adhesion molecule 1	*ICAM1*
24	Induced myeloid leukemia cell differentiation protein Mcl-1	*MCL1*
25	Interferon gamma	*IFNG*
26	Glutathione S-transferase P	*GSTP1*
27	CD40 ligand	*CD40LG*
28	Hepatocyte growth factor receptor	*MET*
29	Apoptosis regulator Bcl-2	*BCL2*
30	Apoptosis regulator BAX	*BAX*
31	Interleukin-1 beta	*IL1B*
32	C-C motif chemokine 2	*CCL2*
33	Vascular cell adhesion protein 1	*VCAM1*
34	Interleukin-8	*CXCL8*
35	Myeloperoxidase	*MPO*
36	Nuclear factor erythroid 2-related factor 2	*NFE2L2*
37	C-reactive protein	*CRP*
38	C-X-C motif chemokine 10	*CXCL10*
39	Osteopontin	*SPP1*
40	Glutathione S-transferase Mu 1	*GSTM1*
41	Leukocyte elastase	*SERPINB1*
42	Matrix metalloproteinase 1	*MMP1*
43	Matrix metalloproteinase 7	*MMP7*
44	Matrix metalloproteinase 12	*MMP12*
45	Phosphatidylinositol-4,5-bisphosphate 3-kinase catalytic subunit, gamma isoform	*PIK3CG*
46	Carbonic anhydrase II	*CA2*
47	Tyrosine-protein kinase receptor UFO	*AXL*
48	Vascular endothelial growth factor receptor 2	*KDR*
49	Tyrosine-protein kinase SRC	*SRC*
50	Beta-glucuronidase	*GUSB*
51	Glutathione S-transferase A1	*GSTA1*
52	Focal adhesion kinase 1	*PTK2*
53	Androgen receptor	*AR*
54	Trypsin-1	*PRSS1*
55	Dipeptidyl peptidase IV	*DPP4*
56	Caspase-9	*CASP9*
57	Mitogen-activated protein kinase 1	*MAPK1*
58	Cellular tumor antigen p53	*TP53*
59	NF-kappa-B inhibitor alpha	*NFKBIA*
60	Xanthine dehydrogenase/oxidase	*XDH*
61	DNA topoisomerase 1	*TOP1*
62	E3 ubiquitin-protein ligase Mdm2	*MDM2*
63	Proliferating cell nuclear antigen	*PCNA*
64	Heme oxygenase 1	*HMOX1*
65	Baculoviral IAP repeat-containing protein 5	*BIRC5*
66	Interleukin-2	*IL2*
67	G2/mitotic-specific cyclin-B1	*CCNB1*
68	Interleukin-4	*IL4*
69	Insulin receptor	*INSR*
70	Serotonin transporter	*SLC6A4*
71	Cytochrome P450 2C19	*CYP2C19*
72	Butyrylcholinesterase	*BCHE*
73	Cytochrome P450 17A1	*CYP17A1*
74	Nuclear receptor ROR-gamma	*RORC*
75	Fatty acid-binding protein, liver	*FABP1*
76	Phospholipase A2 group 1B	*PLA2G1B*
77	CD81 antigen	*CD81*
78	UDP-glucuronosyltransferase 2B7	*UGT2B7*
79	Mitogen-activated protein kinase 8	*MAPK8*
80	Signal transducer and activator of transcription 1-alpha/beta	*STAT1*
81	Cytochrome P450 3A4	*CYP3A4*
82	Cytochrome P450 1A2	*CYP1A2*
83	Cytochrome P450 1A1	*CYP1A1*
84	Matrix metalloproteinase 3	*MMP3*
85	Epidermal growth factor receptor	*EGF*
86	ETS domain-containing protein Elk-1	*ELK1*
87	Ornithine decarboxylase	*ODC1*
88	Caspase-8	*CASP8*
89	Superoxide dismutase [Cu-Zn]	*SOD1*
90	Protein kinase C alpha type	*PRKCA*
91	Hypoxia-inducible factor 1-alpha	*HIF1A*
92	Myc proto-oncogene protein	*MYC*
93	Tissue factor	*F3*
94	Protein kinase C beta type	*PRKCB*
95	Heat shock protein beta-1	*HSPB1*
96	Tissue-type plasminogen activator	*PLAT*
97	Thrombomodulin	*THBD*
98	Interleukin-1 alpha	*IL1A*
99	Neutrophil cytosol factor 1	*NCF1*
100	Poly [ADP-ribose] polymerase 1	*PARP1*
101	C-X-C motif chemokine 11	*CXCL11*
102	C-X-C motif chemokine 2	*CXCL2*
103	Inhibitor of nuclear factor kappa-B kinase subunit alpha	*CHUK*
104	Cathepsin D	*CTSD*
105	Interferon regulatory factor 1	*IRF1*
106	Receptor tyrosine-protein kinase erbB-3	*ERBB3*
107	Serum paraoxonase/arylesterase 1	*PON1*
108	Muscarinic acetylcholine receptor M3	*CHRM3*
109	Protein kinase C delta	*PRKCD*
110	Heparin cofactor 2	*SERPIND1*
111	Transcription factor Jun	*JUN*
112	Cyclin-dependent kinase 1	*CDK1*
113	Polyunsaturated fatty acid 5-lipoxygenase	*ALOX5*
114	Tyrosine-protein phosphatase non-receptor type 11	*PTPN11*
115	Catalase	*CAT*
116	Mu-type opioid receptor	*OPRM1*
117	Solute carrier family 22 member 12	*SLC22A12*
118	Casein kinase II subunit alpha	*CSNK2A1*
119	Neutrophil elastase	*ELANE*
120	Tyrosine-protein kinase Lck	*LCK*
121	Tyrosine-protein kinase SYK	*SYK*
122	Transthyretin	*TTR*
123	Cystic fibrosis transmembrane conductance regulator	*CFTR*
124	ATP-dependent translocase ABCB1	*ABCB1*
125	Maltase-glucoamylase	*MGAM*
126	Amyloid-beta precursor protein	*APP*
127	ADP-ribosyl cyclase/cyclic ADP-ribose hydrolase 1	*CD38*
128	Macrophage migration inhibitory factor	*MIF*
129	Vasopressin V2 receptor	*AVPR2*
130	Thrombin	*F2*
131	Insulin-like growth factor-binding protein 2	*IGFBP2*
132	C-X-C chemokine receptor type 1	*CXCR1*
133	Immunoglobulin heavy constant gamma 1	*IGHG1*
134	Leukotriene A-4 hydrolase	*LTA4H*
135	Aldo-keto reductase family 1 member B1	*AKR1B1*
136	Protein c-Fos	*FOS*
137	Cyclin-dependent kinase inhibitor 2A	*CDKN2A*
138	Phosphatidylinositol 3,4,5-trisphosphate 3-phosphatase and dual-specificity protein phosphatase PTEN	*PTEN*
139	Collagen alpha-1	*COL3A1*
140	Growth arrest-specific protein 6	*GAS6*

### 3.3 Construction and analysis of PPI network

**FIGURE 4:**
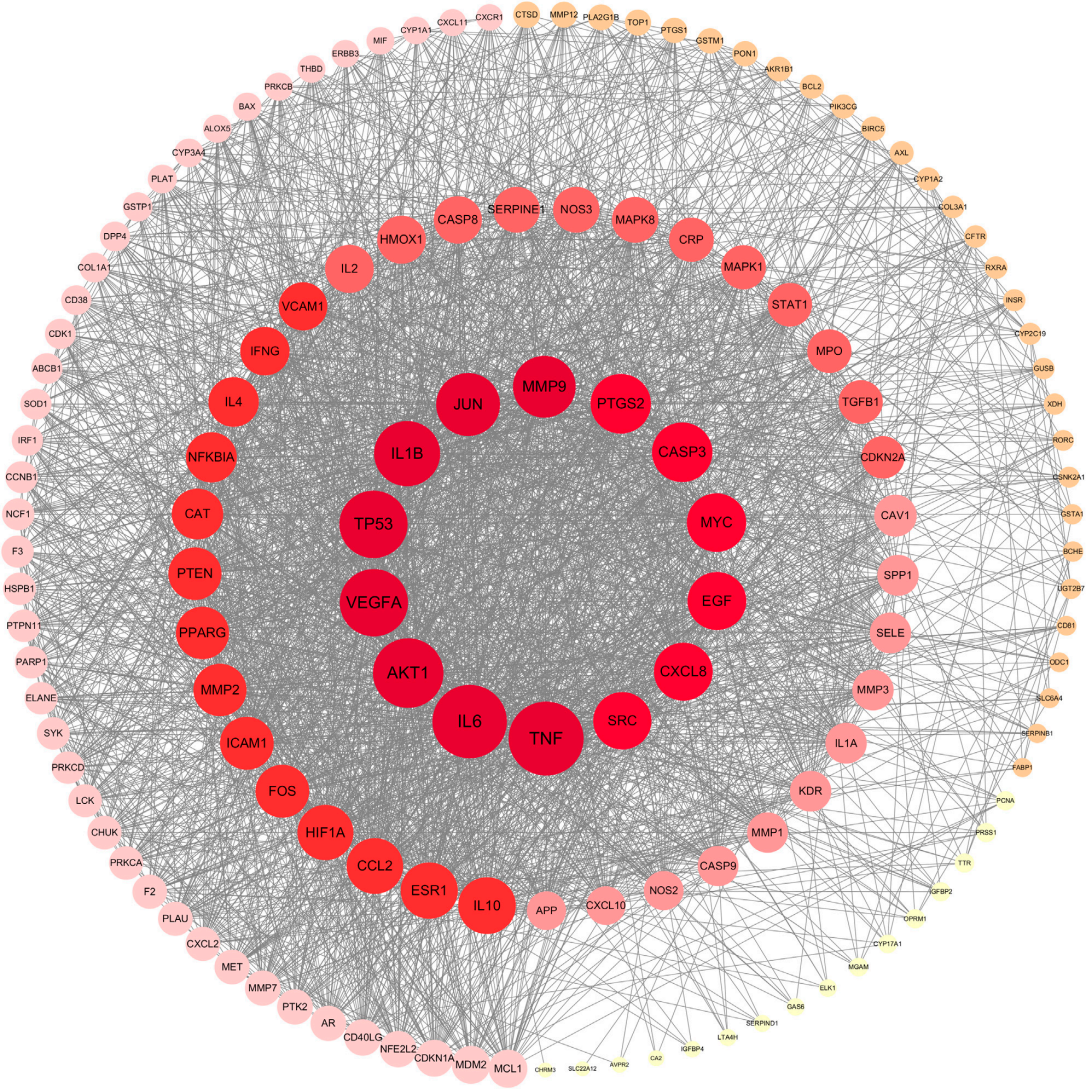
PPI network. Nodes are colored from light to dark. The darker the color is, the larger the degree value of the target is, and the more important the target is in the network.

The PPI network, containing 140 nodes and 2,660 edges, was constructed using the STRING database and visualized by the Cytoscape software (Figure 4). In this network, the node represented the active compound of SHP and the compound-related target, and the edge represented interactions among active compounds and target proteins. Additionally, the node size is proportional to the target degree value. The higher the degree value, the larger the node and the more important it is. Afterwards, targets were sorted according to degree values, and the first 10 key targets with the highest degree value were listed in Table 4. The first 10 targets were used to identify the active ingredients of herbs they corresponded to, and TNF, AKT1 and PTGS2 with a largest number of corresponding active ingredients were regarded as key targets in the study. Meanwhile, the top three components corresponding to abundant core targets were quercetin, kaempferol and luteolin, which were the main active components.

**TABLE 4:** Ten important protein targets with top degree of PPI network.

Number	Protein target name	Degree
1	TNF	108
2	IL6	107
3	AKT1	100
4	VEGFA	96
5	TP53	96
6	IL1B	92
7	JUN	88
8	MMP9	86
9	PTGS2	82
10	CASP3	82

### 3.4 Construction of herb-compound-target-nephritis network

**FIGURE 5:**
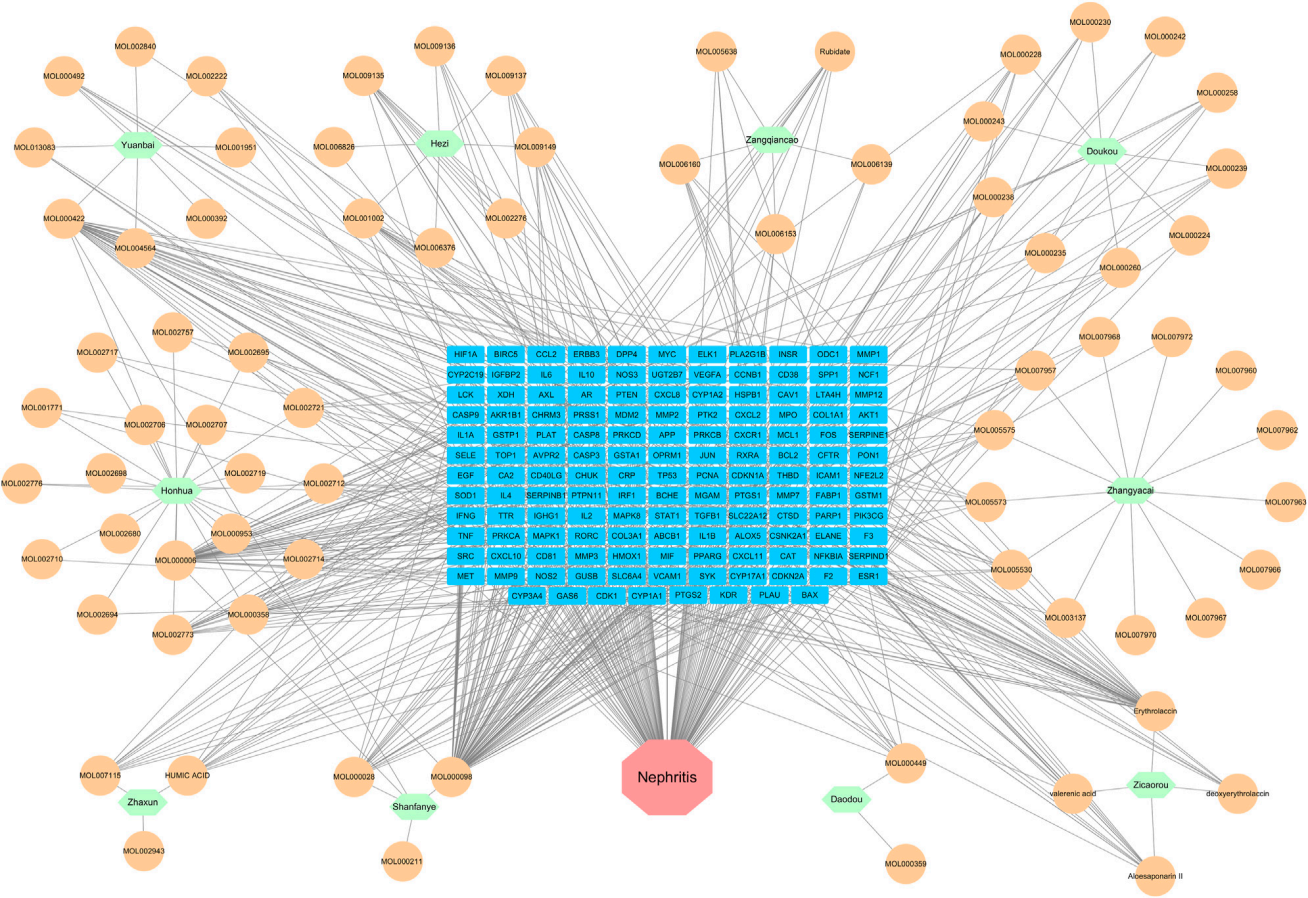
Herb-ingredient-target-nephritis network. Green represents herbs, orange represents ingredients, blue represents targets, and red represents diseases.

As illustrated in Figure 5, an herb-compound-target-nephritis network was constructed to provide a clearing visualization of the relationship among herbs, ingredients, targets and nephritis. In this network, green denotes herbs, orange denotes ingredients, blue denotes targets, and red denotes diseases.

### 3.5 GO and KEGG analyses

**FIGURE 6:**
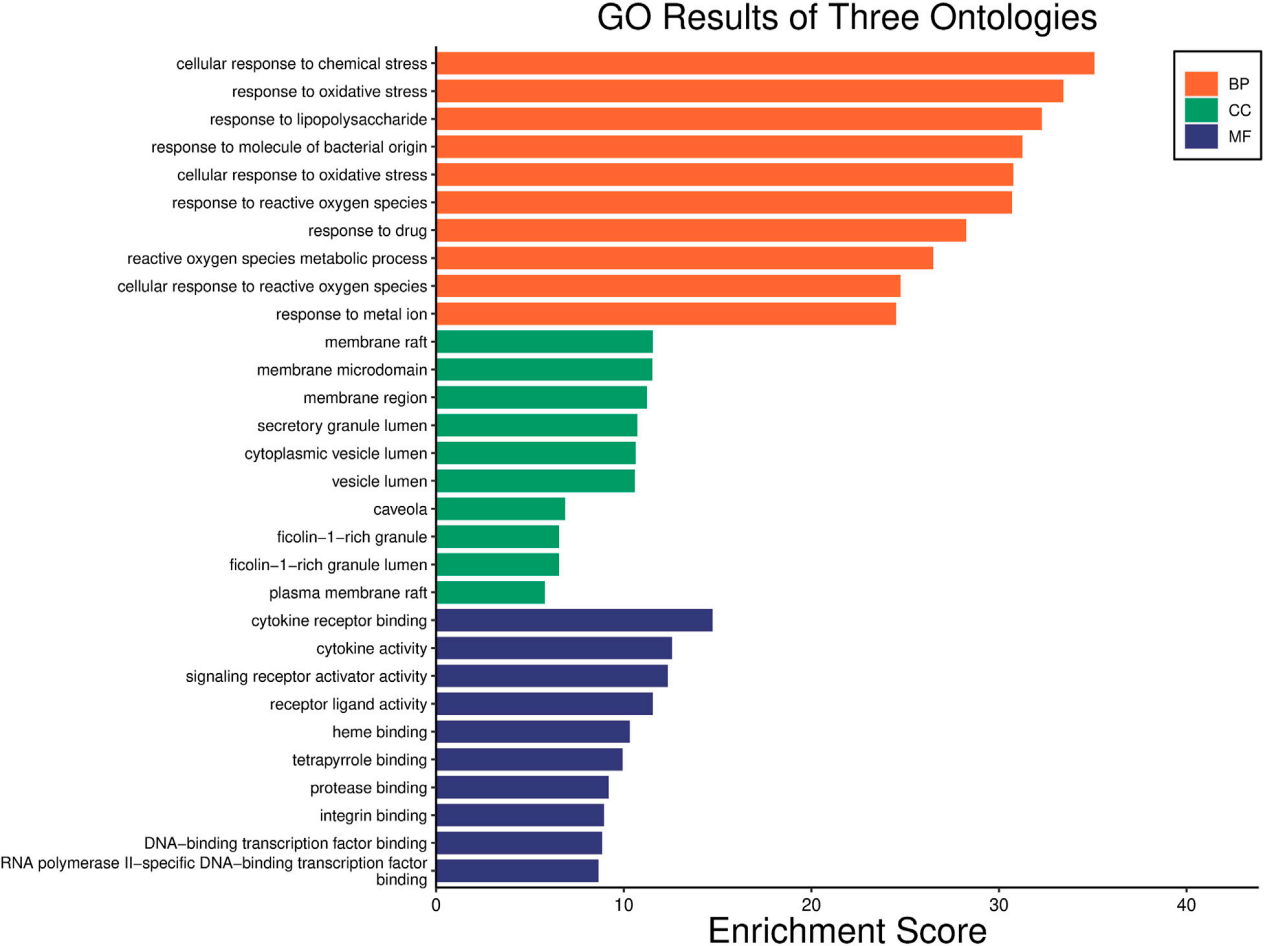
GO analysis of common targets. The *y*-axis shows three categories associated with targets, such as “Biological Process” (BP) categories, “Cellular component” (CC) categories, and “Molecular function” (MF) categories; the *x*-axis shows the enrichment scores of these terms. The length of the column reflects *p*-value [-log10 (*p*-value)].

**FIGURE 7:**
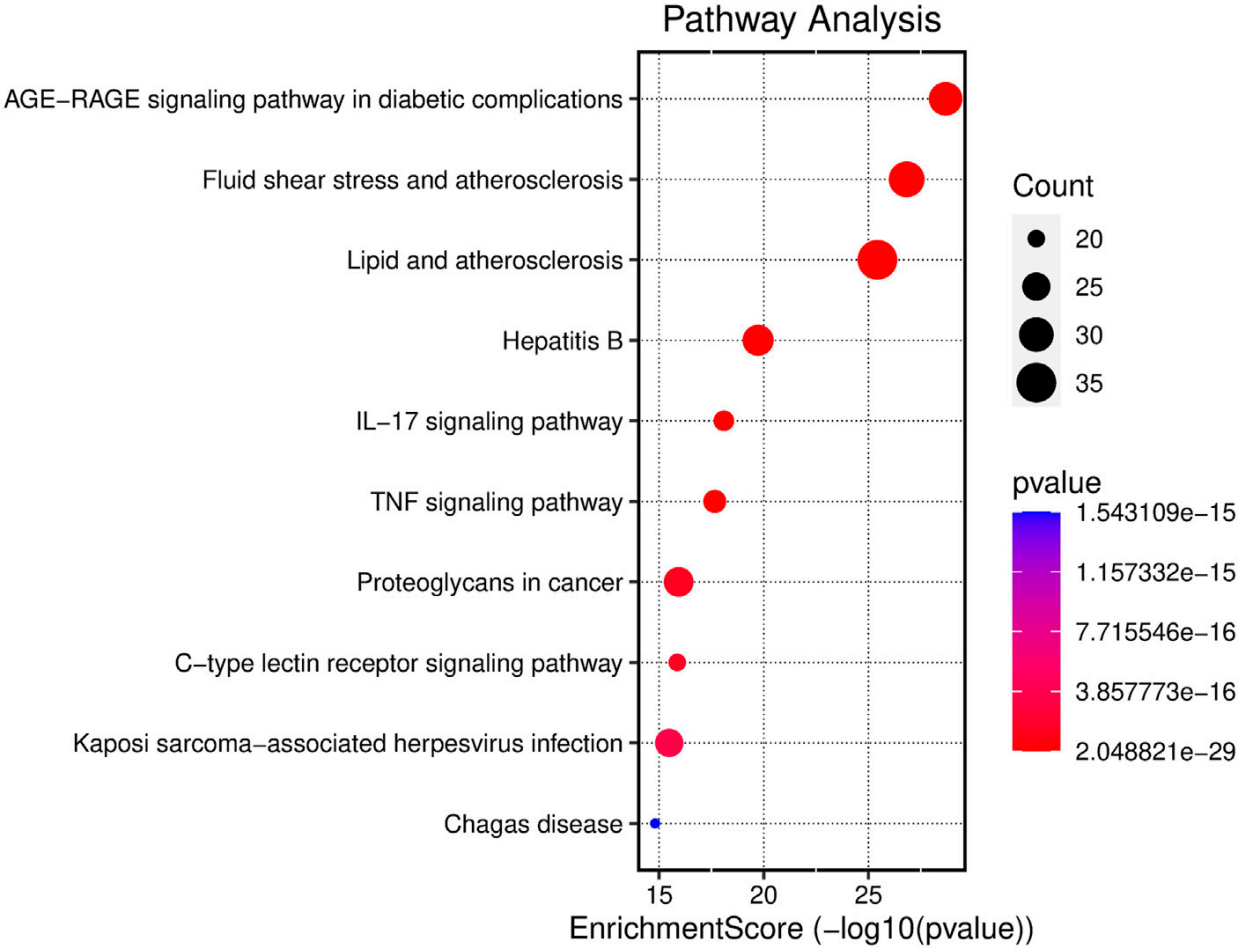
Enrichment analysis of KEGG pathway in SHP anti-nephritis. The area of the bubble represents the number of enriched genes in the pathway, and the color of the bubble represents the size of the *p*-value.

**FIGURE 8:**
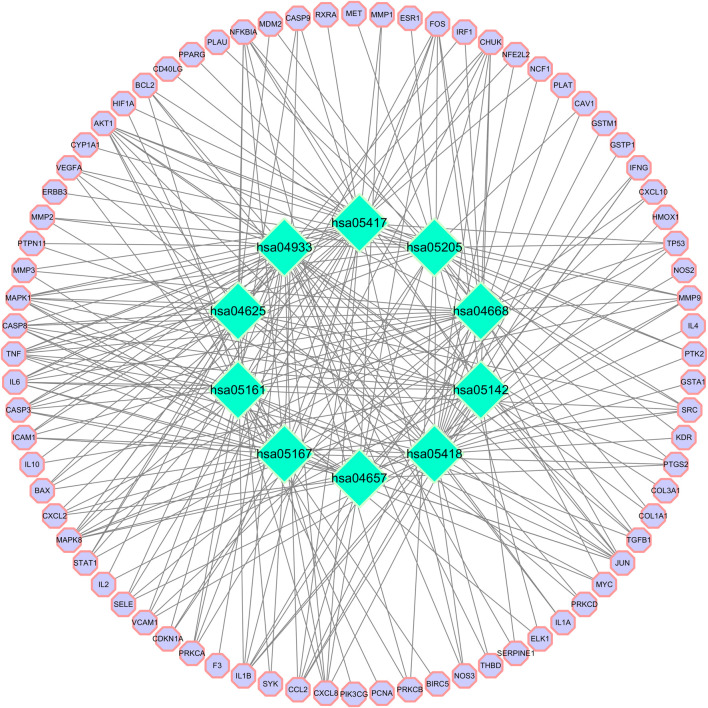
Target-pathway network. Green represents the pathway. The purple circle is the target. Hsa04933: AGE-RAGE signaling pathway in diabetic complications; hsa05418: Fluid shear stress and atherosclerosis; hsa05417: Lipid and atherosclerosis; hsa05161: Hepatitis B; hsa04657: IL-17 signaling pathway; hsa04668: TNF signaling pathway; hsa05205: Proteoglycans in cancer; hsa04625: C-type lectin receptor signaling pathway; hsa05167: Kaposi sarcoma-associated herpesvirus infection; and hsa05142: Chagas disease.

GO functional enrichment analysis of target genes in the PPI network was performed using the Bioinformatics website, and a total of 2,163 entries (2,014 of the BP category, 61 of the CC category, and 143 of the MF category) were provided. The top 10 significant enrichment results of each category are shown in Figure 6. The BP entries were mainly related to cell responses to chemical/oxidative stress and lipopolysaccharide. The CC entries focused on membrane rafts, membrane microdomains and membrane regions. The MF entries covered cytokine receptor binding, cytokine activity, and signaling receptor activator activity. Subsequently, the KEGG pathway enrichment analysis was conducted on 140 common targets, and 186 regulated pathways (Figure 7), such as the AGE-RAGE signaling pathway in diabetic complications, the IL-17 signaling pathway and the TNF signaling pathway, were identified. In addition, a target pathway network was established based on the first 10 paths in Figure 7, as shown in Figure 8.

### 3.6 Molecular docking of ingredients and targets

**FIGURE 9:**
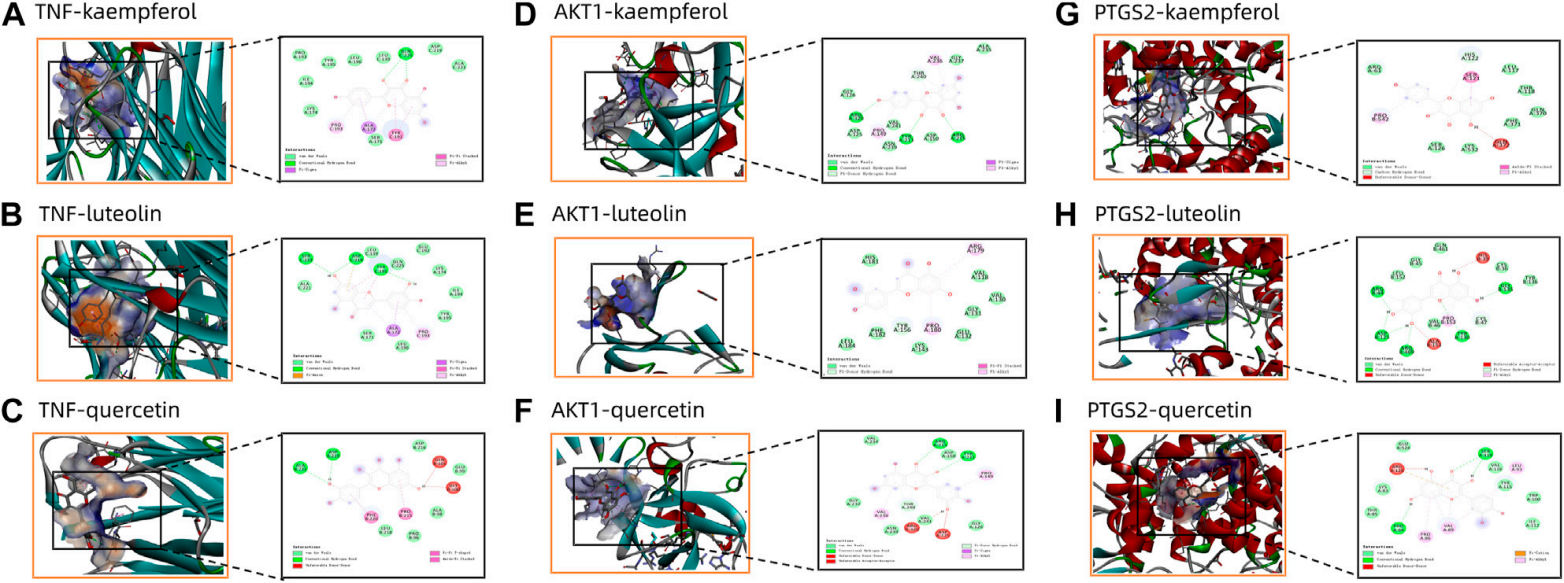
Panel **(A–I)** shows the molecular docking diagram of quercetin, kaempferol and luteolin with TNF, AKT1 and PTGS2.

The binding interactions between main active ingredients (quercetin, kaempferol and luteolin) and key targets (TNF, AKT1 and PTGS2) was verified by molecular docking. The molecular docking binding performance is presented in Table 5. The interaction patterns of these three ingredients with key targets are shown in Figure 9. It is generally believed that binding energy less than −4.25 kcal/mol, −5.0 kcal/mol or −7.0 kcal/mol indicates certain, good or strong binding activity between ligand and receptor, respectively. Therefore, our results indicated a stable complex consisting of quercetin, kaempferol and luteolin, and these three ingredients all showed a strong binding activities with TNF, AKT1 and PTGS2 (binding energy < −6.9 kcal/mol).

**TABLE 5:** The binding energy values of quercetin, kaempferol and luteolin with TNF, AKT1 and PTGS2.

Molecule ID	Compound	Target protein	PDB identifier	Estimated ΔG (kcal/mol)
MOL000098	Quercetin	TNF	7jra	−6.9
		AKT1	3os5	−7.5
		PTGS2	5f19	−7.1
MOL000422	Kaempferol	TNF	7jra	−7.3
		AKT1	3os5	−7.6
		PTGS2	5f19	−8.4
MOL000006	Luteolin	TNF	7jra	−7.4
		AKT1	3os5	−6.8
		PTGS2	5f19	−9.6

## 4 Discussion

Nephritis is characterized by various pathological forms and clinically presents with albuminuria, hematuria, hypertension and edema. If left untreated, it can lead to renal shrinkage and decreased function. Therefore, it is urgent to develop safer and more effective anti-nephritis drugs (Chadban and Atkins, 2005). In this study, the active components of SHP and its anti-nephritis molecular mechanism were explored by network pharmacology.

In this study, quercetin, kaempferol and luteolin had higher target frequency, suggesting that they played a central role in the treatment of nephritis. Quercetin, with anti-inflammatory, antioxidant and neuroprotective propertie, is a natural flavonoid found in a wide range of fruits, herbs and vegetabless (Shen, Lin et al., 2021). A study has suggested that quercetin can help ameliorate lupus nephritis (LN)-associated renal fibrosis and inflammation (Chen, Chiang et al., 2022). Kaempferol is a dietary flavonoid existed in various plants (Wong, Chin et al., 2019) and has been explored to have protective effects on the kidneys of rats with radiation nephritis (Mostafa, Edmond et al., 2022). Luteolin is another kind of flavonoids commonly found in medicinal plants, and exhibits a strong anti-inflammatory activity both *in vitro* and *in vivo* (Aziz, Kim et al., 2018). Kin et al. had probed that luteolin may be able to mitigate kidney inflammation and interstitial fibrosis (Kim, Kim et al., 2016). Collectively, quercetin, kaempferol and luteolin in SHP may all play a role in the treatment of nephritis.

By mapping SHP and nephritis-related targets, 140 shared genes between SHP and nephritis were detected. In order to further understand the interaction among these gene-encoded proteins, a PPI network was constructed. The results showed that TNF, AKT1 and PTGS2 were the main targets of SHP in treating nephritis, which was consistent with previous reports. The *TNF* gene encodes a multifunctional proinflammatory cytokine belonging to the tumor necrosis factor superfamily (Wu, Wen et al., 2020). Studies (Bantis, Heering et al., 2006) have revealed that the G-308A polymorphism of the *TNF-*α gene is associated with the expression of the −308A allele and the increase of TNF-α production, making TNF a risk factor for membranous glomerulonephritis (Müller, Hoppe et al., 2019). AKT encodes one of three members of the human AKT serine-threonine protein kinase family, and it is commonly referred to as the protein kinases B encoding one of three key component of many signaling pathways. It has been demonstrated that subcellular C5b-9 complex can induce the proliferation of glomerular mesangial cells in rat Thy-1 glomerulonephritis by activating *TRAF6*-mediated *PI3K*-dependent *AKT1* (Qiu, Zhang et al., 2012). PTGS2 (also known as *COX-2*), a prostaglandin endoperoxidase, exerts a key effect in prostaglandin biosynthesis. It has been reported that there is a certain relationship between *COX-2* inhibitors and acute interstitial nephritis (Albrecht, Giebel et al., 2017). Juan Jin et al. (Jin, Lin et al., 2018) have revealed that the over-expression of *COX-2* can lead to renal autophagy and injury. Thus, the regulation of TNF, AKT1 and PTGS2 may contribute to the treatment of nephritis.

After identifying the main targets (TNF, AKT1 and PTGS2) of SHP in nephritis treatment, we further conducted a KEGG analysis to reveal the signal pathways of these main targets. The results showed that the AGE-RAGE, IL-17 and TNF signal pathways were the important pathways of SHP acting on nephritis. Studies have shown that the accumulation of *AGE* and *RAGE* in the kidneys and other tissues of diabetic patients is related to the development of diabetic nephropathy and vascular diseases (Tanji, Markowitz et al., 2000). Additionally, a study on the pathogenesis of LN has pointed out that AGE-RAGE can regulate high nitrotyrosination in LN, thereby reducing the oxidative stress in LN (Ene, Georgescu et al., 2021). IL-17 has also been recognized as an independent risk factor for LN prognosis and an effective indicator for the clinical diagnosis, treatment and prognosis of LN (Paquissi and Abensur, 2021). Similarly, multiple evidences have suggested that recently discovered T cells (Th17 cells) that produce interleukin 17 (IL-17) are involved in the renal inflammatory cascade associated with glomerulonephritis (Ramani and Biswas, 2016). The TNF signaling pathway is also related to nephritis. For instance, Xiaoping Qing et al. have elucidated that the TNF signaling pathway plays a key role in irreversible LN kidney damage (Qing, Chinenov et al., 2018). Moreover, it has been found that TNF-α production in T lymphocytes alleviates NTN-induced kidney injury and fibrosis by inhibiting renal T helper 17 lymphocyte response and neutrophil infiltration (Wen, Rudemiller et al., 2020). In conclusion, we speculateed that SHP may ameliorate nephritis by regulating the AGE-RAGE, IL-17 and TNF signaling pathways.

To further verify the relationship between active ingredients (quercetin, kaempferol and luteolin) and key targets (TNF, AKT1 and PTGS2), we carried out molecular docking. The results showed that the binding energies of quercetin and luteolin with TNF, AKT1 and PTGS2 were lower than −5.0 kJ/mol, indicating the potential for forming an effective and stable complex between the ligand and receptor. In short, the core active compounds in SHP appeared to regulate SHP-related pathways by acting on the important genes linked to nephritis, thus offering therapeutic value for nephritis treatment.

However, our research has some limitations. Bioactive ingredients of SHP were screened only from existing public databases and literatures, rather than using mass spectrometry and other methods. Additionally, there is a lack of animal experiments and clinical trials to verify our findings. To further improve our research, more animal experiments and clinical trials will be conducted in the future.

## 5 Conclusion

In this study, we combined network pharmacology and molecular docking to explore the mechanism by which SHP exerts its anti-nephritis effects. We found that quercetin, kaempferol and luteolin are likely the main active compounds of SHP responsible for its therapeutic effects against nephritis. Moreover, SHP can target the expression of TNF, AKT1 and PTGS2 via the AGE-RAGE, IL-17 and TNF signaling pathways. Overall, although more researches are needed to clarify the exact mechanism, this study provides a valuable insight into the application of SHP for nephritis treatment and the potential for future anti-nephritis drug development.

## Data Availability

The datasets presented in this study can be found in online repositories. The names of the repository/repositories and accession number(s) can be found in the article/supplementary material.
